# Tumor-derived exosomes RNA expression profiling identifies the prognosis, immune characteristics, and treatment in HR+/HER2-breast cancer

**DOI:** 10.18632/aging.204986

**Published:** 2023-08-29

**Authors:** Qi Zhou, Jing Wang, Haiping Zhang, Lu Sun, Jingjing Liu, Lingchao Meng, Jingwu Li

**Affiliations:** 1 Department of Breast Surgery, People’s Hospital of Tangshan, Tangshan 063001, Hebei, China; 2 Department of Breast Center, People’s Hospital of Tangshan, Tangshan 063001, Hebei, China; 3 Department of Oncology, Tangshan People’s Hospital, Tangshan 063001, Hebei, China; 4 Department of Pathology, Tangshan People’s Hospital, Tangshan 063001, Hebei, China; 5 The Cancer Institute, Tangshan People’s Hospital, Tangshan 063001, Hebei, China

**Keywords:** exosomes, breast cancer, immune characteristics, prognosis, endocrine therapy

## Abstract

Exosomes play crucial roles in intercellular communication and are involved in the onset and progression of various types of cancers, including breast cancer. However, the RNA composition of breast cancer-derived exosomes has not been comprehensively explored. We conducted microarray assays on exosomes isolated from breast cancer and healthy breast epithelial cells from three patients with hormone receptor (HR) +/ human epidermal growth factor receptor (HER2) - breast cancer and identified 817 differentially expressed genes (DEGs). Among these, 315 upregulated tumor-derived exosome genes (UTEGs) were used to classify HR+/HER2- breast cancers into two categories, revealing a difference in survival rates between the groups. We developed and validated a novel prognostic exosome score (ES) model consisting of four UTEGs that provides a refined prognosis prediction in HR+/HER2-breast cancer. ES reflects various immune-related features, including somatic variation, immunogenicity, and tumor immune infiltrate composition. Our findings indicate a considerable positive correlation between the ES and drug sensitivity values for vincristine, paclitaxel, and docetaxel. However, ES was remarkably higher in the endocrine therapy non-responder group than in the responder group. Immunohistochemistry confirmed the remarkable expression of the four model genes in tumor tissues, and their expression in MCF-7 cell exosomes was higher than that in MCF10A cells, as verified via qPCR. In summary, tumor-derived exosome genes provide novel insights into the subtyping, prognosis, and treatment of HR+/HER2-breast cancer.

## INTRODUCTION

Breast cancer (BRCA) is a frequently diagnosed malignancy that affects both sexes and remains the leading cause of cancer-related deaths in females [1]. It is projected by the American Cancer Society that by 2023, the United States will see 290,560 new cases of breast cancer and 43,780 fatalities [2]. BRCA can be classified into four molecular subtypes based on the hormone receptor (HR) and human epidermal growth factor receptor 2 (HER2) status: HR+/HER2-, HR+/HER2+, HR-/HER2+, and TNBC (HR-/HER2-). Each subtype exhibits distinct biological features, with HR+/HER2- BRCA being the most common subtype that can be managed using endocrine therapy [3]. However, despite the high sensitivity of HR+/HER2- BRCA to endocrine therapies, a considerable proportion of patients develop resistance to these interventions, with disease recurrence occurring during or after treatment [4].

In the 1970s, Johnstone initially characterized exosomes as 30-150 nm vesicles composed of a phospholipid bilayer membrane. Exosomes vehicles (EVs) arise through inward invagination of the endosomal membrane, enabling them to transfer information from their originating cells. These vesicles contain an array of molecular elements, such as DNA, RNA, proteins, lipids, and metabolites, which reflect the cell types from which they are derived. Exosomes have been implicated in drug resistance, cancer progression, and metastasis. Campos et al. reported the presence of Caveolin-1 in EVs from the metastatic breast cancer cell line MDA-MB-231, promoting the *in vitro* migration and invasion of the same cells, as well as a non-metastatic breast cancer cell line [5]. Furthermore, Semina et al. showed that co-culturing sensitive MCF-7 cells with exosomes from drug-resistant cells for 14 days induced sensitivity to antiestrogen drugs [6]. Exosomes have drawn increasing attention since they emerged as critical players in the initiation, progression, and metastasis of breast cancer [7]. Nonetheless, there are still inadequate data regarding the RNA content of breast cancer-derived exosomes.

**Figure 1:**
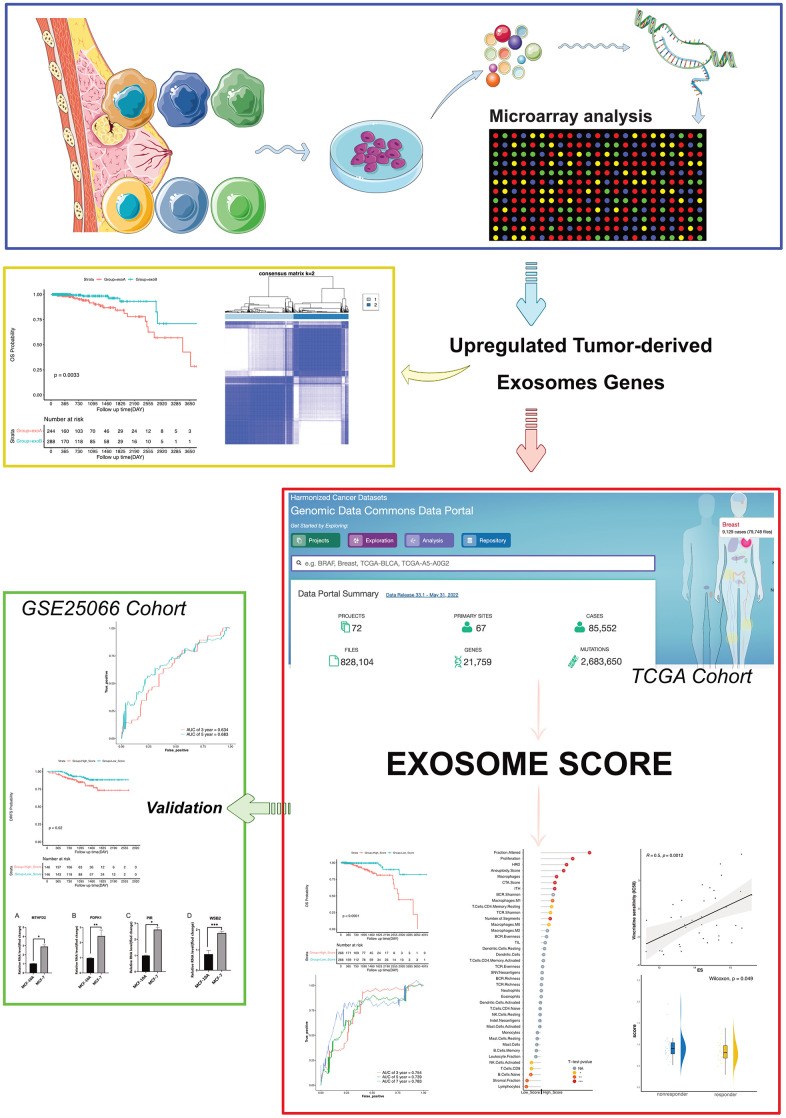
Strategy for identifying upregulated tumor-derived exosomes genes and exosomes score in this study.

In our study, we recruited patients who had been diagnosed with HR+/HER2- BRCA. Exosomes were extracted from primary cultured healthy and cancerous breast epithelial cells, and their gene expression was scrutinized via microarray. Differentially expressed genes (DEGs) were identified via bioinformatics analysis, and we formulated predictive models and exosome scores (ES) for patients with breast cancer based on the expression of four genes, phosphoinositide-dependent protein kinase 1 (*PDPK1*), WD Repeat and SOCS Box Containing 2 (*WSB2*), pirin (*PIR*), and Methylenetetrahydrofolate Dehydrogenase 2 (*MTHFD2*). Furthermore, it was discovered that the ES not only exhibited a correlation with survival but also mirrored the status of diverse immune-related traits and the effectiveness of endocrine therapy in patients with breast cancer (Figure 1).

## MATERIALS AND METHODS

### Tissue collection and public data sets

From February to March 2022, a cohort of consecutive patients with early-stage breast cancer was enrolled at the Tangshan People’s Hospital. These patients were required to meet all the following inclusion criteria:1) age between 55 and 65 years with no prior history of breast cancer or other malignancy and without any past chemotherapy treatment, 2) confirmed diagnosis of breast cancer through needle biopsy histology, and 3) no history of medical intervention for breast cancer prior to surgical resection. The following exclusion criteria were applied: inflammatory breast cancer, hypertension (blood pressure > 140/90 mmHg), hyperlipidemia (triglyceride > 1.7 mmol/L, total cholesterol > 6.0 mmol/L), diabetes, and tumors with a diameter larger than 5 cm.

Carcinoma and healthy tissue samples were obtained from surgical specimens immediately after surgery. Fresh tissues were used to prepare single-cell suspensions and subsequent primary cultures, which were then frozen and stored at -80° C for later analysis.

In The Cancer Genome Atlas (TCGA) Breast Cancer (BRCA) project, masked copy number segments, RNASeq expression (STAR – Counts), and clinical data were downloaded using the TGCAbiolinks R package [8]. The TCGA RNA sequencing FPKM data extracted from “STAR – Counts” files were transformed into log2 (FPKM + 1). Survival data and breast cancer subtypes were compiled from clinical data and a total of 532 patients with HR+/HER2-breast cancer with a follow-up time of >30 days were selected (Supplementary Table 4).

The Gene Expression Omnibus (GEO) dataset GSE25066 [9], which includes 292 patients with HR+/HER2- breast cancer with distant relapse-free survival (DRFS) data (Supplementary Table 5), was used for validation.

Dataset GSE145325 [10] contains RNA-seq data of patients with ER+ breast cancer treated with letrozole, and SRR files (SRP249306) were downloaded from Sequence Read Archive (SRA) stores and converted to FASTQ format using the SRAtoolkit.

### Cell lines and cell culture

The human breast epithelial cell line MCF10A was procured from the Peking Union Medical College Cell Resource Center (PUMCCRC) in Beijing, China. The MCF-7 human breast cancer cell line was obtained from the Shanghai Cell Bank of the Chinese Academy of Sciences (CAS). Cells were cultured according to the cell instructions respectively.

### Exosomes isolation

Exosomes present in the supernatants of breast cancer cell cultures were purified using differential ultracentrifugation. Briefly, the cells were grown in cell culture medium supplemented with 10% Fetal Bovine Serum (FBS)-exosomes depleted (SBI, USA) for 48 h. Thereafter, the cell culture supernatant was collected and subjected to centrifugation at 2× 10^3^ g for 20 min at 4° C to eliminate cells. A subsequent centrifugation at 1× 10^4^ g for 30 min at 4° C was performed to remove cellular debris. The resulting supernatant was sieved using 0.2 μm filters (Millipore, USA) and subsequently ultra-centrifuged at 1× 10^5^ g for 1h at 4° C. The pellets were resuspended in phosphate-buffered saline (PBS) and once again subjected to ultra-centrifugation at 1× 10^5^ g for 1h at 4° C.

### Purified exosomes transmission electron microscopy

A volume of 10 μl of exosomes was administered onto copper transmission electron microscopy grids, measuring 3.05 mm with 200 mesh, and left to rest for 5 min. The grids were rinsed with PBS and coated with 2% uranyl acetate for 3 min. Exosome images were captured using a transmission electron microscope (Tecnai G2 Spirit Biotwin; FEI Company, USA).

### RNA isolation and qRT-PCR

Cells total RNA was extracted using Trizol Reagent (Invitrogen) according to the manufacturer’ protocol. Subsequently, 1 μg of RNA was subjected to reverse transcription using the PrimeScript™ RT Master Mix kit (Takara, China). The SYBR premix Ex TaqTM II kit (Takara, China) was used to detect the expression levels of the specified genes, and the results were analyzed using the Stratagene Mx 3000P software (Agilent Technologies, USA). The 2^–ΔΔCt^ method was used to calculate the relative expression levels of mRNA. Primer sequences used in this analysis are listed in Supplementary Table 1. All qRT-PCR experiments were repeated in triplicate, and each group had three technical replicates. Statistical analyses were performed using GraphPad Prism 8.0. The outcomes of the experiments were expressed as the mean ± SD, and a two-tailed Student’s t-test was employed to compute the *p*-value. Statistical significance was considered when *p*-values were <0.05.

### Microarray analysis and data processing

RNA quantity and quality were measured using a NanoDrop ND-1000. RNA integrity was assessed via standard denaturing agarose gel electrophoresis or an Agilent 2100 Bioanalyzer.

Sample labeling and array hybridization were performed according to the Agilent One-Color Microarray-based Gene Expression Analysis protocol (Agilent Technologies). The Agilent Feature Extraction software (version 11.0.1.1) was used to analyze the acquired array images. Long non-coding (lnc) RNAs and mRNAs that were flagged at least in three out of six samples as Present or Marginal (“All Targets Value”) were chosen for further data analysis.

### Microarray probes re-annotation

The Human reference sequence (GRCh38.d1.vd1) and annotation file (GDC.h38 GENCODE v36 GTF) were downloaded from The Genomic Data Commons (GDC) database (https://gdc.cancer.gov/about-data/gdc-data-processing/gdc-reference-files).

We mapped the probe sequences to the human genome (GRCh38.d1.vd1.fa.tar.gz) using the SeqMap software (http://www-personal.umich.edu/~jianghui/seqmap/). Using an annotation file (gencode.v22.annotation.gtf.gz), we reannotated the probes for the two chips and removed those corresponding to multiple genes.

### Identification of prognostic genes and construction of a risk model

Univariate Cox regression analysis was performed to explore the correlation between genes and OS in TCGA dataset. DEGs with *p* <0.05 were considered candidate genes.

Random forest analysis was used to reduce the scope of the gene screening. Finally, the coefficient for each gene was obtained through multivariate Cox regression, and the exosome score (ES) = sum of coefficient × expression level of the gene was calculated.

### Immune-related features and tumor microenvironment data

Thorsson conducted an extensive immunogenic study of more than 10,000 tumors containing 33 different types of cancer using data collected from TCGA [11]. We used Thorsson’s outcomes to investigate the association between ES and immune-related features. Similarly, Tamborero et al. [12] provided data on immune infiltration patterns, which we used to investigate the association between the ES and tumor immune microenvironment. All data were numerical and transformed into standardized z-scores for statistical analysis and presentation.

### Data availability

Microarray data is available in the Gene Expression Omnibus: GSE207304 Source code for model construction is available at https://github.com/QZhou-Ch/BC-Exosomes.

### Statistical analysis

Most statistical analyses were performed using R (version 4.2.0) with default arguments unless mentioned otherwise. The Shapiro–Wilk test was used to test data normality. The Wilcox test was performed to verify the statistical significance between two groups, whereas the Kruskal–Wallis test was applied to test for multiple groups.

Differential expression analysis was performed using the R package DESeq2 [13]. Survival and survminer packages were used for survival analysis, and the time-dependent receiver operating characteristic (ROC) and area under the curve (AUC) were determined using the R package survival ROC. *P*-values were two-sided and adjusted for multiple testing using the Benjamini–Hochberg False Discovery Rate (FDR), and statistical significance was set at *p* < 0.05 or FDR < 0.1.

## RESULTS

### Differential gene expression in exosomes of luminal breast cancer cells and healthy breast cells

**Figure 2:**
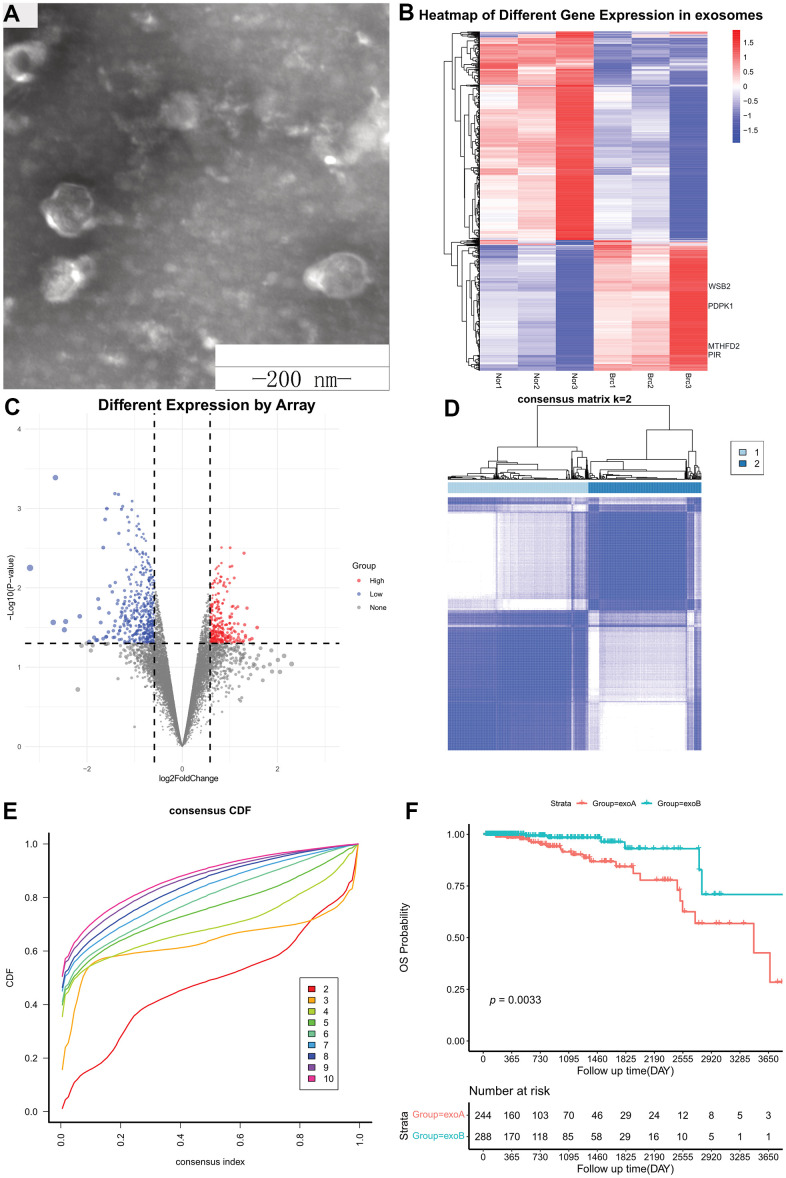
**Differentially expressed genes from exosomes.** (**A**) Exosomes were observed using transmission electron microscopy. Scale bar, 200 nm. (**B**) Heatmap of Different Gene Expression in exosomes. (**C**) Volcano plot of Different Gene Expression in exosomes. (**D**) Consensus clustering matrix for k = 2. (**E**) Consensus clustering CDF for k = 2–10. (**F**) Kaplan–Meier survival curves of OS according to two clusters in TCGA cohort. CDF, cumulative distribution function; OS, overall survival; TCGA, The Cancer Genome Atlas.

Three pairs of breast cancer and healthy breast tissues were collected at the Tangshan People’s Hospital to establish primary cultures (Supplementary Table 2). Subsequently, the supernatant of the cell culture medium was collected, and exosomes were extracted via differential ultracentrifugation (Figure 2A). Total RNA was extracted from exosomes and used to determine gene expression patterns using microarrays. Background correction was performed with the backgroundCorrect function using the “normexp” method, and normalization was performed with the normalizeBetweenArrays function using the “normexp” method. When a gene contained multiple probes, the maximum value was considered as the gene expression value. Differential expression analysis was performed using the R Bioconductor package, limma. DEGs were selected using a fold-change cut-off of >1.5 or < -1.5 and a *p*-value < 0.05. In total, 817 DEGs were identified in cancer vs. healthy exosomes, including 315 upregulated tumor-derived exosomes genes (UTEGs) and 502 downregulated tumor-derived exosomes genes (Figure 2B, 2C).

### UTEGs cluster analysis

We used the 315 UTEGs in patients with HR+/HER2- BRCA from TCGA to perform a consensus cluster analysis (maxK = 10, reps = 1000, clusterAlg = “km,” distance=“euclidean”) and the proportion of ambiguous clustering (PAC) score showed an optimal K of two (Figure 2D, 2E). The 532 patients with breast cancer were divided into two categories according to the optimal K value: ExoA and ExoB, with 244 and 288 cases, respectively. Kaplan–Meier survival curves (Figure 2F) revealed that patients in the ExoA group had poorer survival than those in the ExoB group (HR, 0.286; 95% CI, 0.136-0.600; log-rank *p* = 0.33 e-03). Therefore, we believe that this new type of cluster classification will be beneficial for accurate clinical diagnosis and treatment.

### Development and evaluation of ES in TCGA cohort

To meet the cross-platform availability of the model, we hope that it can be applied to the Affymetrix Human Genome U133A Array platform because it is the most widely used chip with the largest amount of data available. After Univariate Cox analysis of the TCGA dataset, five UTAGs genes were eventually included in the candidate model. Feature importance was determined via random forest survival analysis (Figure 3A, 3B) with default parameters (ntree = 5000), and we used four genes to construct the final model: *PDPK1*, *WSB2*, *PIR*, and *MTHFD2* (details in Supplementary Table 3).

**Figure 3:**
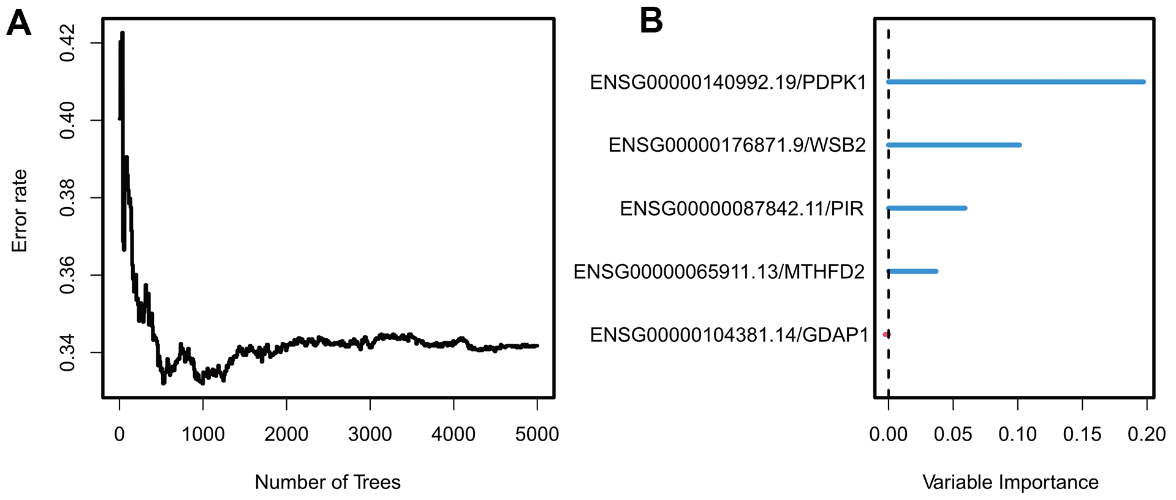
**Construction and validation of exosome scores.** (**A**) The relationship between error rate and the number of trees. (**B**) Random Forest feature importance ranking for the five predictive features.

**Figure 3:**
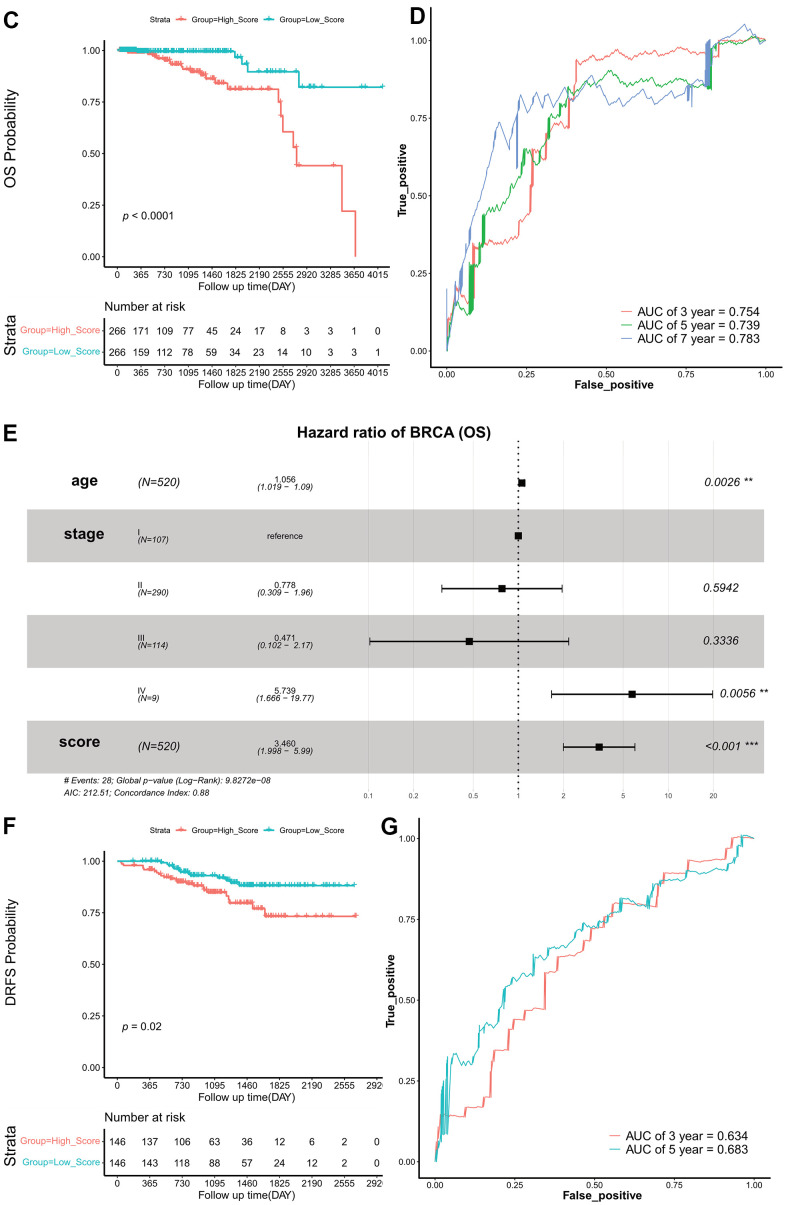
**Construction and validation of exosome scores.** (**C**) Kaplan–Meier survival curves of OS according to ES groups in TCGA cohort. (**D**) ROC curves at 3-, 5-, and 7 years of OS according to ES groups in the TCGA cohort. (**E**) Forest plots of OS. (**F**) Kaplan–Meier survival curves of DRFS according to ES groups in the GEO validation set. (**G**) ROC curves at 3- and 5 years of DRFS according to ES groups in the GEO validation set. ES, exosome score; OS, overall survival; TCGA, The Cancer Genome Atlas; ROC, receiver operating characteristic; AUC, area under the curve; DRFS, distant relapse-free survival; AIC, Akaike information criterion; GEO, Gene Expression Omnibus.

The patients in the TCGA set were split into two groups, high- and low-score groups, according to the median ES. The survival curves (Kaplan–Meier estimates) revealed that the OS in the low-score group was significantly higher than that in the high-score group (HR, 0.170; 95% CI, 0.081–0.359; log-rank *p* = 0.380e-04) (Figure 3C). The AUC of ES for OS were 0.754 at 3 years, 0.739 at 5 years, and 0.783 at 7 years (Figure 3D).

Moreover, to evaluate the independent prognostic effect of ES, we used a multivariate COX regression model to adjust for other factors, including age and TNM stage. Results indicate that ES remained a significant and independent prognostic indicator in TCGA cohort (Figure 3E).

### Prognostic power evaluation of ES in the GEO cohort

To validate the robustness of the risk signature, the ES for each patient with HR+/HER2- BRCA in the GEO cohort was also evaluated. The median ES was used to divide patients into high- and low-score groups. Kaplan–Meier survival curves revealed that DRFS was significantly prolonged in the low-scoring group (HR, 0.4594778; 95% CI, 0.241–0.877; log-rank *p* = 0.020) (Figure 3F). The AUC of IS were 0.634 and 0.683 at three and five years, respectively (Figure 3G).

### Different immune-related features between high- and low score patients in TCGA cohort

**Figure 4:**
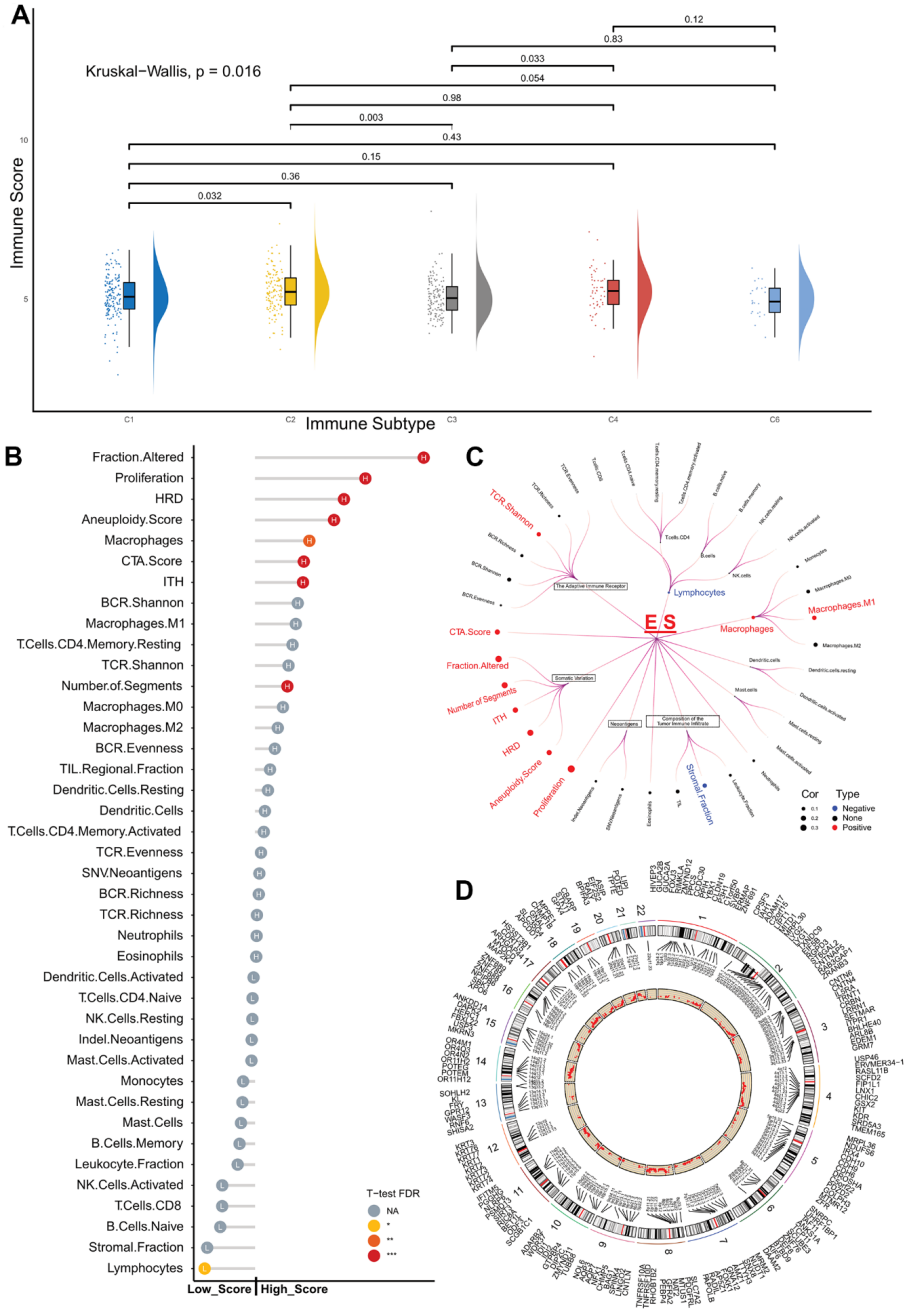
**Exploration of the exosome score predictive role in the TCGA cohort.** (**A**) Raincloud Plot shows the comparison of ES between the different immune subtypes. (**B**) Lollipop plot showing the comparison of immune-related features between the low- and high-score groups. The length of the stick represents the difference between the medians of the features in the high- and low-score groups. (**C**) Circos Plot displays the CNVs distribution in ES-relevant groups, and red dots represent the difference between the incidence of CNVs in the high- and low-score groups. (**D**) Violin plot showing the distribution of the two groups in different immune cells, NA *p* > 0.05, * *p* < 0.05, ** *p* < 0.01, *** *p* < 0.001. ES, exosome score; TCGA, The Cancer Genome Atlas; CNVs, copy number variations; aDC, activated dendritic cells; CD8+ T, CD8 T cells; iDC, immature dendritic cells; NKbright, NK CD56 bright cells; NKdim, NK CD56 dim cells; Tcm, central memory T cells; Tem, effector memory T cells; Tfh, follicular helper T cells; Tgd, gamma delta T cells; Th, helper T cells; Treg, regulatory T cells.

First, we examined the distribution of ES in five immune types in patients with breast cancer. A substantial difference was observed between the five immune subtypes of the Kruskal–Wallis test (*p* = 0.016), as shown in Figure 4A. Furthermore, patients with type C3 (Lymphocyte Depleted) had a lower ES than type C2 (IFN-γ Dominant) and type C4 (Lymphocyte Depleted) subtypes.

In the following analysis, we explored the differences in the composition of the tumor immune infiltrate, somatic variation, immunogenicity, and genomic state between the ES-based subtypes of the TCGA dataset (Figure 4B).

The high-score group had higher proliferation, altered fraction, silent and non-silent mutation rates, as well as a reduced stromal fraction than the low-score group.

Potential factors that presented tumor somatic or germline mutations, including homologous recombination defects, intratumor heterogeneity, number of segments, aneuploidy score, non-silent silent mutation rates, were compared between the high- and low-score groups. The median values for the variables of the high-score group were substantially higher than those of the low-score group.

Finally, given the differences in tumor immune infiltrates between the low- and high-score groups, we analyzed the immune microenvironments in both groups. The Wilcox test revealed that high-scoring patients had significantly higher abundances of central memory T cells (Tcm) and T helper cells (Th), but lower abundances of B cells, immature dendritic cells (iDC), mast cells, neutrophils, NK CD56 bright cells (NKbright), effector memory T cells (Tem), and gamma delta T cells (Tgd) (Figure 4D) than the low-score group.

### CNV incidence between low- and high-score patients in TCGA cohort

We found significant differences between the two groups in tumor somatic or germline mutations, which were both calculated from CNV files; therefore, we performed a statistical analysis of CNVs events in both groups of patients.

The ‘Masked Copy Number Segment File’ and ‘SNP6 GRCH38 Remapped Probeset File for CNV Analysis,’ were used for GISTIC2 analysis [14] on the GenePatter website [15]. The CNV values were further thresholder using a noise cutoff of 0.3. A chi-square test was performed to confirm the difference in focal CNVs events between the high- and low-score groups.

Patients in the high-score group had a higher incidence of CNVs events in 6861 genes than those in the low-score group (FDR < 0.1) (Figure 4C) (Supplementary Table 6).

### ES can reflect the treatment sensitivity

We further identified associations between the model and the treatment effects of chemotherapeutic agents and endocrine therapy. The Genomics of Drug Sensitivity in Cancer (GDSC) database was adopted to evaluate the correlation between the ES and drug sensitivity values (IC50) of molecules in 50 breast cell lines (Tissue = “breast”). Consequently, vincristine, paclitaxel, and docetaxel, which target microtubules, appeared to be significantly and positively correlated with ES (Figure 5A–5C). Other common chemotherapeutic drugs, such as doxorubicin and cisplatin; and endocrine therapy drugs, such as tamoxifen and fulvestrant, were not significantly associated with ES (*p*>0.05, Figure 5D–5G). Tamoxifen, fulvestrant or anastrozole, and exemestane, mechanism lies in reducing the interaction of estrogen with cancer cells. Therefore, we evaluated the relationship between ES and endocrine therapy in another clinical study of letrozole treatment.

**Figure 5:**
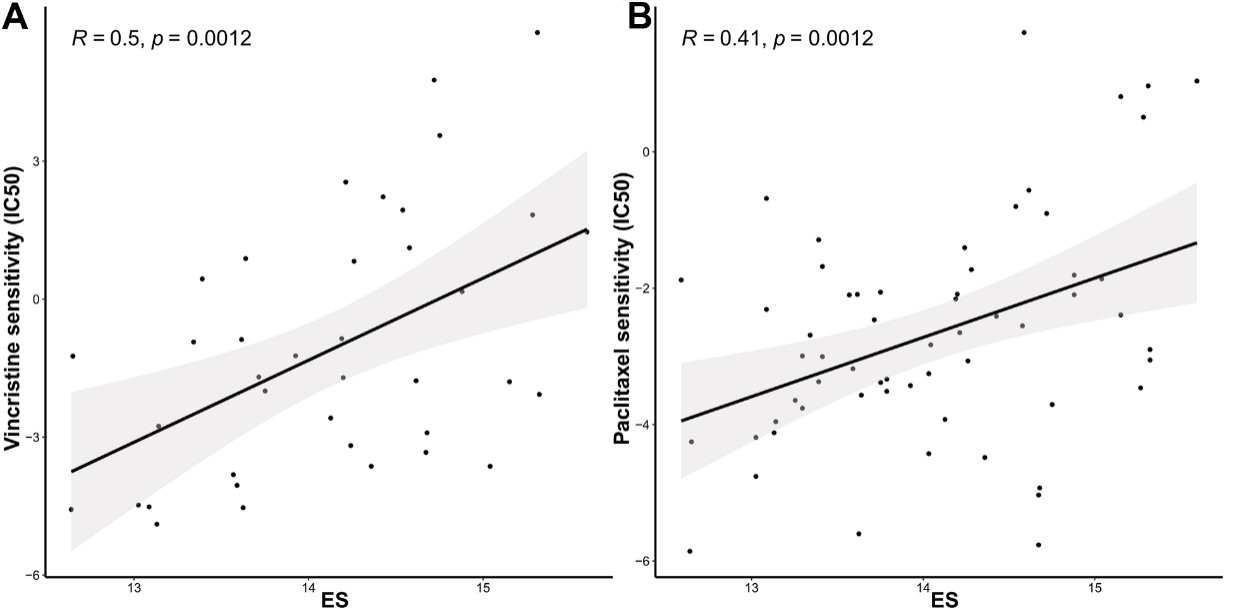
**The ES predicting the sensitivity to chemosensitivity and endocrine therapy.** Scatter plots for associations between drug sensitivity values (IC50) and (**A**) vincristine, (**B**) paclitaxel.

**Figure 5:**
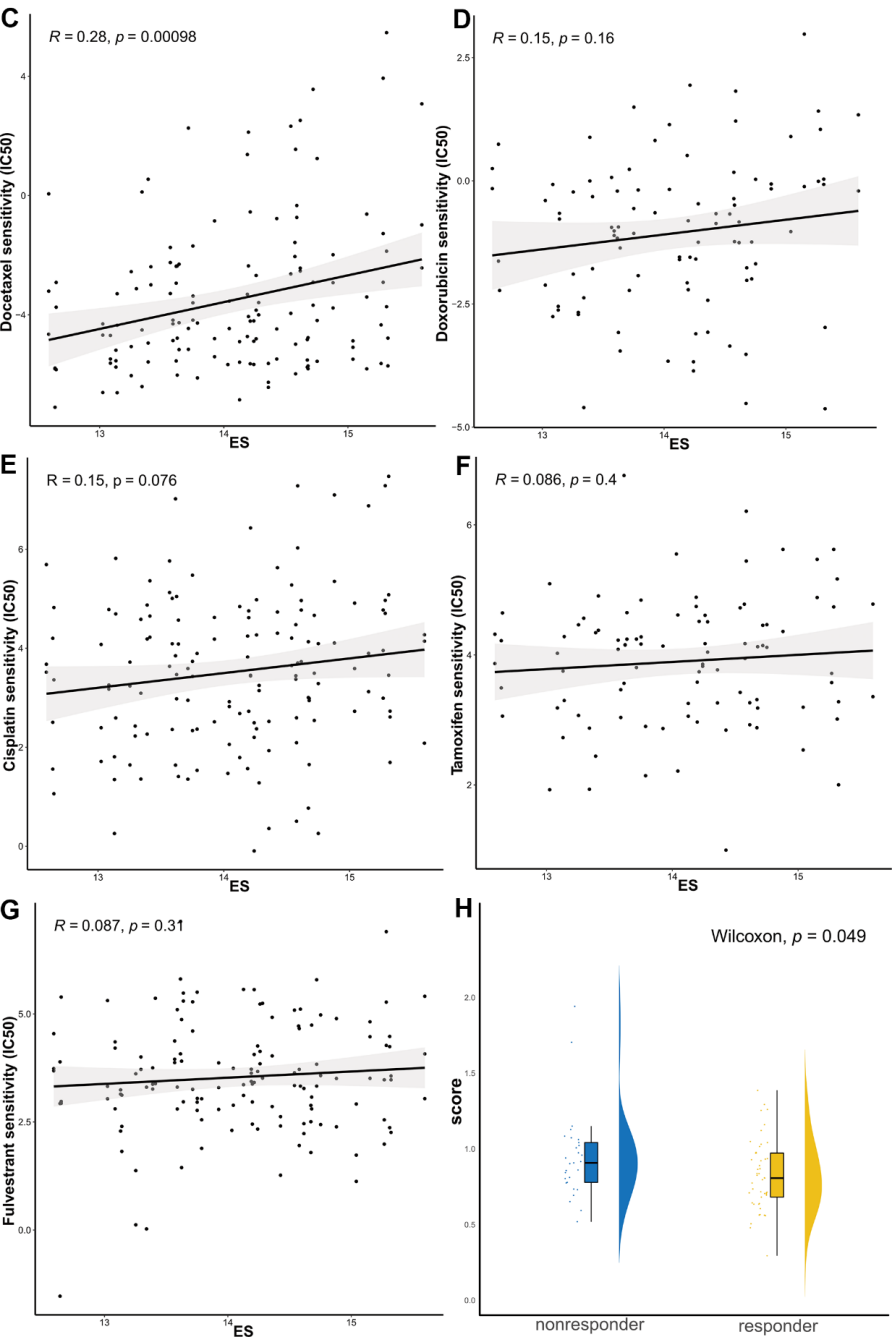
**The ES predicting the sensitivity to chemosensitivity and endocrine therapy.** (**C**) docetaxel, (**D**) doxorubicin, (**E**) cisplatin, (**F**) tamoxifen, and (**G**) fulvestrant; (**H**) Raincloud Plot showing the comparison of ES between endocrine therapy non-responder and responder groups. ES, exosome score; IC50, half maximal inhibitory concentration.

Because the study did not provide the exact reference genome or annotation file, we re-aligned the reads using the fastp-Hisat2-featurecounts pipeline. Based on the counts data (Supplementary Table 7), we calculated FPKM and ES in GSE145325 dataset. The Wilcoxon test revealed that ES in the non-responder group was significantly higher than that in the responder group (Figure 5H).

### Validation of the expression of four tumor-derived exosome genes

**Figure 6:**
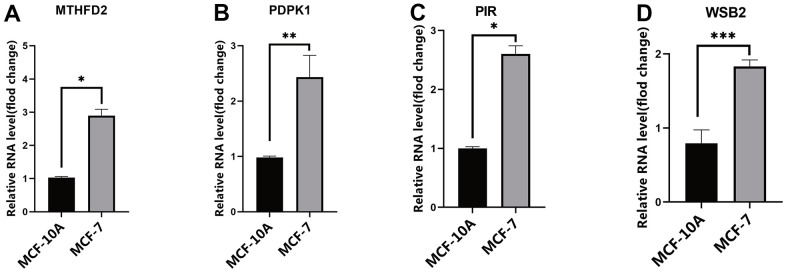
**The expression of risk model genes.** (**A**) MTHFD2 mRNA expression levels in exosomes from MCF10A and MCF-7 cell line; (**B**) PDPK1 mRNA expression levels in exosomes from MCF10A and MCF-7 cell line; (**C**) PIR mRNA expression levels in exosomes from MCF10A and MCF-7 cell line; (**D**) WSB2 mRNA expression levels in exosomes from MCF10A and MCF-7 cell line, * *p* < 0.05, ** *p* < 0.01, *** *p* < 0.001. MTHFD2, methylenetetrahydrofolate dehydrogenase 2; PDPK1, phosphoinositide-dependent protein kinase 1; PIR, pirin; WSB2, WD repeat, and SOCS box containing 2.

The mRNA expression levels of *PDPK1*, *WSB2*, *PIR*, and *MTHFD2* were evaluated in exosomes extracted from the MCF-7 and MCF10A cell lines. The results suggest that the mRNA expression of the four genes was higher in MCF-7 exosomes than in MCF10A exosomes (Figure 6A–6D).

## DISCUSSION

Tumor-derived exosomes that transport RNA have emerged as a major area of research. During the course of our study, we observed insufficient data on HR+/HER2- types of breast cancer. Consequently, we conducted primary cultures of breast cancer and healthy breast epithelial cells from three patients, followed by isolation of the supernatant and RNA extraction from the exosomes. Microarray assays were performed to obtain reliable data for future studies in this domain.

The relationship between gene expression and the prognosis of malignant tumors is currently a hot research topic [16–18], and several gene signatures such as PAM50, Oncotype DX assay (a 21-gene signature), and MammaPrint (a 70-gene signature), which have garnered FDA approval, have been employed for prognostic and diagnostic purposes in the care of patients with breast cancer. In this study, we stratified HR+/HER2- type breast tumors into two categories using 315 UTEGs and the results indicated a disparity in survival between the two groups. Hence, we anticipate that this novel cluster classification will aid future clinical diagnoses and therapies.

To enhance prognostic prediction in breast cancer, we developed a novel prognostic model consisting of four UTEGs. Previous studies have shown that all genes in this signature are considerably associated with breast cancer. *PDPK1*, for instance, is involved in several signaling pathways, including PI3K/Akt, Ras/MAPK, and Myc, which are frequently altered in cancers [19–21].

Maurer et al. [22] identified *PDPK1* overexpression and increased copy number as common events in breast cancer. *PDPK1* enhances the ability of upstream lesions to signal AKT, which accelerates cell growth and migration, rendering the cells more resistant to PI3K inhibition. Recently, the *WSB2* has attracted increasing attention from researchers. Ma et al. [23] found that *WSB2* suppression by shRNA inhibited melanoma cell growth and migration by regulating β-catenin. In breast cancer, overexpression of *WSB2* may promote MCF-7 cell migration, whereas *miR-28-5p* inhibits the migration of breast cancer cells by regulating *WSB2* expression. Thus, the *miR-28-5p/WSB2* axis may represent a novel therapeutic target in breast cancer [24]. Pirin, a transcriptional coregulator of NF-kappa-B, has also been identified as a critical gene in our model. It facilitates the binding of NF-kappa-B proteins to target kappa-B genes in a redox-state-dependent manner [25, 26]. Knockdown of *PIR* in MCF7 and MDA-MB-231 cell lines caused a dramatic decrease in cell proliferation and xenograft tumor growth in mice by activating *E2F1* and its target genes [27]. *MTHFD2* encodes a mitochondrial enzyme involved in folate metabolism that is induced in multiple tumors to meet the high biosynthetic demand for cell proliferation [28]. Suppression of *MTHFD2* in MCF-7 cells resulted in altered levels of intracellular serine and glycine and an increase in glycolytic activity [29–31].

Accumulating evidence suggests that exosomes released from cancer cells play remarkable roles in promoting proliferation, immune regulation, chemoresistance, and carcinogenesis [32]. Multiple studies have demonstrated that tumor-derived exosomes can induce cell proliferation. Al-Nedawi showed that microvesicles derived from glioblastoma cells carry the oncogenic receptor EGFRvIII, which actively promotes tumor cell proliferation and invasion [33]. Moreover, exosomes from tumor cells participate in the immune response. A survey found that exosomes produced by breast cancer cells in the circulatory system activate macrophages through NF-kappa-B signaling, and over-produce various inflammatory cytokines to induce proinflammatory activity [34]. Recent evidence also indicates that exosomes play a crucial role not only in regulating drug resistance but also in transferring drug resistance to drug-sensitive BRCA cells. Chemotherapy induces the secretion of multiple extracellular vesicles encapsulating miRNAs, such as *miRNA-9-5p*, *miRNA195-5p*, and *miRNA-203a-3p*, which concurrently target the transcription factor One Cut Homeobox 2, leading to the adaptation of cancer stem-like cell traits. Conversely, the downregulation of these miRNAs or the upregulation of One Cut Homeobox 2 expression abolished the cancer stem-like cell-activating effect of extracellular vesicles from chemotherapy-treated BRCA cells [35]. In the present study, we constructed an exosome signature based on breast cancer-derived exosomal genes. We queried relevant studies and found that the four genes included in the model played different roles in various types of cancer cell exosomes [36, 37]. Analysis of the TCGA dataset revealed that the high-score group had a higher incidence of CNV events and proliferation than the low-score group, as well as a different distribution of tumor microenvironment (TME).

According to the NCCN guidelines, endocrine therapy is the preferred adjuvant systemic treatment for patients with stage I HR +/HER2-breast cancer without high-risk factors. However, patients with stage II/III disease, particularly those with high-risk factors, require further adjuvant chemotherapy. Our study revealed that ES is positively correlated with sensitivity to cytotoxic anticancer agents that target microtubules; however, it does not reflect the efficacy of anthracyclines, platinum-based drugs, or endocrine therapeutics. Adjuvant endocrine therapy for breast cancer primarily comprises of selective estrogen receptor modulators (SERMs) and aromatase inhibitors (AIs). The former acts as an estrogen antagonist in cancer cells by binding to estrogen receptors (ER), whereas the latter reduces estrogen levels in postmenopausal women. As these two drugs do not directly kill cancer cells, cellular drug sensitivity tests cannot accurately reflect the impact of endocrine therapy. Therefore, we examined the data from a clinical study and found that ES was greater in patients who were resistant to endocrine therapy. This implies that ES can predict the efficacy of endocrine therapy.

## CONCLUSIONS

Using microarray assays, we identified differentially expressed genes in HR+/HER2-breast cancer exosomes and evaluated four of them to develop a predictive model. The model can predict the prognosis of patients with HR+/HER2-breast cancer and the efficacy of chemotherapy and endocrine therapy, giving us a deeper understanding of HR+/HER2-breast cancer.

## Supplementary Material

Supplementary Tables 1-3

Supplementary Table 4

Supplementary Table 5

Supplementary Table 6

Supplementary Table 7

## References

[1] Sung H, Ferlay J, Siegel RL, Laversanne M, Soerjomataram I, Jemal A, Bray F. Global Cancer Statistics 2020: GLOBOCAN Estimates of Incidence and Mortality Worldwide for 36 Cancers in 185 Countries. CA Cancer J Clin. 2021; 71:209–49. 10.3322/caac.21660 33538338

[2] Siegel RL, Miller KD, Fuchs HE, Jemal A. Cancer statistics, 2022. CA Cancer J Clin. 2022; 72:7–33. 10.3322/caac.21708 35020204

[3] Zhou Q, Zhang HP, Zhao YT, Wang XH, Xiong W, Liu YJ, Zhang JH, and Chinese Society of Breast Surgery. Multi-center investigation of the clinical and pathological characteristics of inflammatory breast cancer based on Chinese Society of Breast Surgery (CSBrs-007). Chin Med J (Engl). 2020; 133:2552–7. 10.1097/CM9.0000000000001104 32925286 PMC7722570

[4] Fillon M. Breast cancer recurrence risk can remain for 10 to 32 years. CA Cancer J Clin. 2022; 72:197–9. 10.3322/caac.21724 35285943

[5] Campos A, Salomon C, Bustos R, Díaz J, Martínez S, Silva V, Reyes C, Díaz-Valdivia N, Varas-Godoy M, Lobos-González L, Quest AF. Caveolin-1-containing extracellular vesicles transport adhesion proteins and promote malignancy in breast cancer cell lines. Nanomedicine (Lond). 2018; 13:2597–609. 10.2217/nnm-2018-0094 30338706

[6] Semina SE, Scherbakov AM, Vnukova AA, Bagrov DV, Evtushenko EG, Safronova VM, Golovina DA, Lyubchenko LN, Gudkova MV, Krasil’nikov MA. Exosome-Mediated Transfer of Cancer Cell Resistance to Antiestrogen Drugs. Molecules. 2018; 23:829. 10.3390/molecules23040829 29617321 PMC6017149

[7] Qiu P, Guo Q, Yao Q, Chen J, Lin J. Characterization of Exosome-Related Gene Risk Model to Evaluate the Tumor Immune Microenvironment and Predict Prognosis in Triple-Negative Breast Cancer. Front Immunol. 2021; 12:736030. 10.3389/fimmu.2021.736030 34659224 PMC8517454

[8] Mounir M, Lucchetta M, Silva TC, Olsen C, Bontempi G, Chen X, Noushmehr H, Colaprico A, Papaleo E. New functionalities in the TCGAbiolinks package for the study and integration of cancer data from GDC and GTEx. PLoS Comput Biol. 2019; 15:e1006701. 10.1371/journal.pcbi.1006701 30835723 PMC6420023

[9] Hatzis C, Pusztai L, Valero V, Booser DJ, Esserman L, Lluch A, Vidaurre T, Holmes F, Souchon E, Wang H, Martin M, Cotrina J, Gomez H, et al. A genomic predictor of response and survival following taxane-anthracycline chemotherapy for invasive breast cancer. JAMA. 2011; 305:1873–81. 10.1001/jama.2011.593 21558518 PMC5638042

[10] Lee KM, Guerrero-Zotano AL, Servetto A, Sudhan DR, Lin CC, Formisano L, Jansen VM, González-Ericsson P, Sanders ME, Stricker TP, Raj G, Dean KM, Fiolka R, et al. Proline rich 11 (PRR11) overexpression amplifies PI3K signaling and promotes antiestrogen resistance in breast cancer. Nat Commun. 2020; 11:5488. 10.1038/s41467-020-19291-x 33127913 PMC7599336

[11] Thorsson V, Gibbs DL, Brown SD, Wolf D, Bortone DS, Ou Yang TH, Porta-Pardo E, Gao GF, Plaisier CL, Eddy JA, Ziv E, Culhane AC, Paull EO, et al, and Cancer Genome Atlas Research Network. The Immune Landscape of Cancer. Immunity. 2018; 48:812–30.e14. 10.1016/j.immuni.2018.03.023 29628290 PMC5982584

[12] Tamborero D, Rubio-Perez C, Muiños F, Sabarinathan R, Piulats JM, Muntasell A, Dienstmann R, Lopez-Bigas N, Gonzalez-Perez A. A Pan-cancer Landscape of Interactions between Solid Tumors and Infiltrating Immune Cell Populations. Clin Cancer Res. 2018; 24:3717–28. 10.1158/1078-0432.CCR-17-3509 29666300

[13] Love MI, Huber W, Anders S. Moderated estimation of fold change and dispersion for RNA-seq data with DESeq2. Genome Biol. 2014; 15:550. 10.1186/s13059-014-0550-8 25516281 PMC4302049

[14] Mermel CH, Schumacher SE, Hill B, Meyerson ML, Beroukhim R, Getz G. GISTIC2.0 facilitates sensitive and confident localization of the targets of focal somatic copy-number alteration in human cancers. Genome Biol. 2011; 12:R41. 10.1186/gb-2011-12-4-r41 21527027 PMC3218867

[15] Reich M, Liefeld T, Gould J, Lerner J, Tamayo P, Mesirov JP. GenePattern 2.0. Nat Genet. 2006; 38:500–1. 10.1038/ng0506-500 16642009

[16] Chu PY, Tzeng YDT, Tsui KH, Chu CY, Li CJ. Downregulation of ATP binding cassette subfamily a member 10 acts as a prognostic factor associated with immune infiltration in breast cancer. Aging (Albany NY). 2022; 14:2252–67. 10.18632/aging.203933 35247251 PMC8954971

[17] Chen X, Wang Y, Li Y, Liu G, Liao K, Song F. Identification of immune-related cells and genes in the breast invasive carcinoma microenvironment. Aging (Albany NY). 2022; 14:1374–88. 10.18632/aging.203879 35120331 PMC8876920

[18] Yu H, Fu Y, Tang Z, Jiang L, Qu C, Li H, Tan Z, Shu D, Peng Y, Liu S. A novel pyroptosis-related signature predicts prognosis and response to treatment in breast carcinoma. Aging (Albany NY). 2022; 14:989–1013. 10.18632/aging.203855 35085103 PMC8833126

[19] Chinen Y, Kuroda J, Shimura Y, Nagoshi H, Kiyota M, Yamamoto-Sugitani M, Mizutani S, Sakamoto N, Ri M, Kawata E, Kobayashi T, Matsumoto Y, Horiike S, et al. Phosphoinositide protein kinase PDPK1 is a crucial cell signaling mediator in multiple myeloma. Cancer Res. 2014; 74:7418–29. 10.1158/0008-5472.CAN-14-1420 25269480

[20] Raimondi C, Falasca M. Targeting PDK1 in cancer. Curr Med Chem. 2011; 18:2763–9. 10.2174/092986711796011238 21568903

[21] Gagliardi PA, Puliafito A, Primo L. PDK1: At the crossroad of cancer signaling pathways. Semin Cancer Biol. 2018; 48:27–35. 10.1016/j.semcancer.2017.04.014 28473254

[22] Maurer M, Su T, Saal LH, Koujak S, Hopkins BD, Barkley CR, Wu J, Nandula S, Dutta B, Xie Y, Chin YR, Kim DI, Ferris JS, et al. 3-Phosphoinositide-dependent kinase 1 potentiates upstream lesions on the phosphatidylinositol 3-kinase pathway in breast carcinoma. Cancer Res. 2009; 69:6299–306. 10.1158/0008-5472.CAN-09-0820 19602588 PMC2727605

[23] Zhang Y, Li Z, Zhao W, Hu H, Zhao L, Zhu Y, Yang X, Gao B, Yang H, Huang Y, Song X. WD repeat and SOCS box containing protein 2 in the proliferation, cycle progression, and migration of melanoma cells. Biomed Pharmacother. 2019; 116:108974. 10.1016/j.biopha.2019.108974 31103822

[24] Ma L, Zhang Y, Hu F. miR-28-5p inhibits the migration of breast cancer by regulating WSB2. Int J Mol Med. 2020; 46:1562–70. 10.3892/ijmm.2020.4685 32945370 PMC7447326

[25] Suleman M, Tahir Ul Qamar M, Saleem S, Ahmad S, Ali SS, Khan H, Akbar F, Khan W, Alblihy A, Alrumaihi F, Waseem M, Allemailem KS. Mutational Landscape of Pirin and Elucidation of the Impact of Most Detrimental Missense Variants That Accelerate the Breast Cancer Pathways: A Computational Modelling Study. Front Mol Biosci. 2021; 8:692835. 10.3389/fmolb.2021.692835 34262943 PMC8273169

[26] Liu F, Rehmani I, Esaki S, Fu R, Chen L, de Serrano V, Liu A. Pirin is an iron-dependent redox regulator of NF-κB. Proc Natl Acad Sci USA. 2013; 110:9722–7. 10.1073/pnas.1221743110 23716661 PMC3683729

[27] Suleman M, Chen A, Ma H, Wen S, Zhao W, Lin D, Wu G, Li Q. PIR promotes tumorigenesis of breast cancer by upregulating cell cycle activator E2F1. Cell Cycle. 2019; 18:2914–27. 10.1080/15384101.2019.1662259 31500513 PMC6791709

[28] Koufaris C, Gallage S, Yang T, Lau CH, Valbuena GN, Keun HC. Suppression of MTHFD2 in MCF-7 Breast Cancer Cells Increases Glycolysis, Dependency on Exogenous Glycine, and Sensitivity to Folate Depletion. J Proteome Res. 2016; 15:2618–25. 10.1021/acs.jproteome.6b00188 27315223

[29] Sugiura A, Andrejeva G, Voss K, Heintzman DR, Xu X, Madden MZ, Ye X, Beier KL, Chowdhury NU, Wolf MM, Young AC, Greenwood DL, Sewell AE, et al. MTHFD2 is a metabolic checkpoint controlling effector and regulatory T cell fate and function. Immunity. 2022; 55:65–81.e9. 10.1016/j.immuni.2021.10.011 34767747 PMC8755618

[30] Nilsson R, Jain M, Madhusudhan N, Sheppard NG, Strittmatter L, Kampf C, Huang J, Asplund A, Mootha VK. Metabolic enzyme expression highlights a key role for MTHFD2 and the mitochondrial folate pathway in cancer. Nat Commun. 2014; 5:3128. 10.1038/ncomms4128 24451681 PMC4106362

[31] Zhang F, Wang D, Li J, Su Y, Liu S, Lei QY, Yin M. Deacetylation of MTHFD2 by SIRT4 senses stress signal to inhibit cancer cell growth by remodeling folate metabolism. J Mol Cell Biol. 2022; 14:mjac020. 10.1093/jmcb/mjac020 35349697 PMC9335224

[32] Li L, Zhao J, Zhang Q, Tao Y, Shen C, Li R, Ma Z, Li J, Wang Z. Cancer Cell-Derived Exosomes Promote HCC Tumorigenesis Through Hedgehog Pathway. Front Oncol. 2021; 11:756205. 10.3389/fonc.2021.756205 34692546 PMC8529041

[33] Al-Nedawi K, Meehan B, Micallef J, Lhotak V, May L, Guha A, Rak J. Intercellular transfer of the oncogenic receptor EGFRvIII by microvesicles derived from tumour cells. Nat Cell Biol. 2008; 10:619–24. 10.1038/ncb1725 Erratum in: Nat Cell Biol. 2008; 10:752. 18425114

[34] Chow A, Zhou W, Liu L, Fong MY, Champer J, Van Haute D, Chin AR, Ren X, Gugiu BG, Meng Z, Huang W, Ngo V, Kortylewski M, Wang SE. Macrophage immunomodulation by breast cancer-derived exosomes requires Toll-like receptor 2-mediated activation of NF-κB. Sci Rep. 2014; 4:5750. 10.1038/srep05750 25034888 PMC4102923

[35] Shen M, Dong C, Ruan X, Yan W, Cao M, Pizzo D, Wu X, Yang L, Liu L, Ren X, Wang SE. Chemotherapy-Induced Extracellular Vesicle miRNAs Promote Breast Cancer Stemness by Targeting *ONECUT2*. Cancer Res. 2019; 79:3608–21. 10.1158/0008-5472.CAN-18-4055 31118200 PMC8972808

[36] Kalra H, Drummen GPC, Mathivanan S. Focus on Extracellular Vesicles: Introducing the Next Small Big Thing. Int J Mol Sci. 2016; 17:170. 10.3390/ijms17020170 26861301 PMC4783904

[37] Hong BS, Cho JH, Kim H, Choi EJ, Rho S, Kim J, Kim JH, Choi DS, Kim YK, Hwang D, Gho YS. Colorectal cancer cell-derived microvesicles are enriched in cell cycle-related mRNAs that promote proliferation of endothelial cells. BMC Genomics. 2009; 10:556. 10.1186/1471-2164-10-556 19930720 PMC2788585

