# Supplementary material for: Tumor-derived exosomes RNA expression profiling identifies the prognosis, immune characteristics, and treatment in HR+/HER2-breast cancer
Source: Aging (Albany NY). 2023 Aug 29;15(16):8471–86. doi: 10.18632/aging.204986 (PMC10497011; doi:10.18632/aging.204986)
Supplement: Supplementary Tables 1-3 [file aging-15-204986-s001.pdf]

SUPPLEMENTARY TABLES

Supplementary Table 1. Primer sequences.

β-actin:
Forward primer: CTGGCCGGGACCTGACT
Reverse primer: TCCTTAATGTCACGCACGATTT
PDPK1
Forward primer: GGAACAGCGCAGTACGTTTCT
Reverse primer: CTCGTTTCCAGCTCGGAATGG
WSB2
Forward primer: TTGCTACGGGACTCAACGATG
Reverse primer: GTGACGCGGAGACCAAAATCA
PIR
Forward primer: GAGCAGTCGGAAGGGGTTG
Reverse primer: TTAACTCGGGTCTGCCAATGC
MTHFD2
Forward primer: AGGACGAATGTGTTTGGATCAG
Reverse primer: GGAATGCCAGTTCGCTTGATTA

Supplementary Table 2. Clinical characteristics of enrolled patients.

	recoded ID	Age	Type	Grade	Maximum tumor diameter	Number of lymph node metastases	Immunohistochemistry			
					(Measured according to pathology)	(Measured according to pathology)	ER	PR	Her2	Ki-67
patient 1	565774	62	Invasive ductal carcinoma	II	2.0cm	0	100%+	100%+	0	10%
patient 2	565297	59	Invasive ductal carcinoma	II	1.9cm	0	90%+	80%+	0	20%
patient 3	563841	60	Invasive ductal carcinoma	II	1.9cm	0	60%+	90%+	1+	5%

Supplementary Table 3. Model genes and their coefficients.

gene symbol	gene name	coef
MTHFD2	ENSG00000065911.13	0.10523284074475
PIR	ENSG00000087842.11	0.479944592959224
PDPK1	ENSG00000140992.19	0.8754054718031
WSB2	ENSG00000176871.9	0.423554406918477
